# Exploring the role of esketamine in alleviating depressive symptoms in mice via the PGC-1α/irisin/ERK1/2 signaling pathway

**DOI:** 10.1038/s41598-023-43684-9

**Published:** 2023-10-03

**Authors:** Na Hu, Xuemei Chen, Chengchuan Chen, Xing Liu, Peng Yi, Tianhao Xu, Jing Jia, Jianguo Feng, Chunxiang Zhang, Xiaobin Wang

**Affiliations:** 1 Department of Anesthesiology, The Affiliated Hospital of Southwest Medical University, Luzhou, 646000 Sichuan Province China; 2 Anesthesiology and Critical Care Medicine Key Laboratory of Luzhou, The Affiliated Hospital of Southwest Medical University, Luzhou, 646000 Sichuan Province China

**Keywords:** Neuroscience, Psychology

## Abstract

Esketamine provides an immediate and noticeable antidepressant effect, although the underlying molecular processes are yet unclear. Irisin induced by aerobic exercise has been implicated in the alleviation of depressive symptoms, whether irisin expression responds to the administration of esketamine remains unknown. In this study, we found that irisin was reduced in the hippocampus and peripheral blood of chronic unpredictable mild stress (CUMS) mice, whereas the irisin level was rescued by esketamine treatment. The reduction of PGC-1α expression (transcriptional regulator of irisin gene expression) in the CUMS mice was rescued by esketamine treatment, PGC-1α knockdown significantly reduced the irisin level induced by esketamine. Additionally, FNDC5/irisin-knockout mice developed more severe depressant-like behaviors than wild-type mice under CUMS stimulation, with an attenuated the antidepressant effect of esketamine. Further research indicated that irisin-mediated modulation of esketamine on depressive-like behaviors in CUMS mice involved the ERK1/2 pathway. Overall, the PGC-1α/irisin/ERK1/2 signaling activation may be a new mechanism underlying the antidepressant activity of esketamine, denoting that irisin may be a promising therapeutic target for the treatment of depression.

## Introduction

Nearly 300 million individuals worldwide are afflicted by the serious mental condition known as major depressive disorder (MDD)^1^. MDD is also the leading cause of suicide, with more than 800,000 deaths annually due to depression^2^. Despite the availability of therapies like pharmacotherapy and cognitive behavioral psychotherapy, a sizable minority of patients continue to be resistant to treatment^3^. These traditional antidepressants often increase a patient’s suicidal ideation on days 1–9 of depression treatment^4^. Thus, drug treatment is not satisfactory overall, and the patient’s quality of life is seriously affected.

Studies have confirmed that, as a non-drug, exercise alleviates depressive symptoms^5^. Such effects are assumed to be mediated by myokines, a group of cytokines and other small proteins secreted by skeletal muscles that contribute to many organ functions^6^. Irisin, a newly discovered myokine, is secreted into circulation from skeletal muscles during exercise from its membrane-bound precursor protein fibronectin type III domain-containing 5 (FNDC5)^7^, and is abundant in various brain areas, most notably the hippocampus and cortex^6^. Several studies have revealed that irisin ameliorates depressive symptoms via modulating energy metabolism, promoting the expression of brain-derived neurotrophic factor (BDNF), and reducing epidermal growth factor receptors^8–10^. In addition to the response to exercise, increasing evidence has shown that drugs affect the expression of irisin in diseases^11–13^. However, the relationship between irisin and antidepressant drugs has not been reported.

Esketamine, the S-enantiomer of ketamine (R,S-ketamine), has a rapid and obvious antidepressant effect^14^. Intranasal esketamine injection is being researched as a therapy for treatment-resistant depression. However, esketamine is similar to ketamine in that it has negative side effects, such as transient dissociative and psychotic symptoms, which make it difficult to take on a regular basis^15^. Hence, the creation of novel treatment targets depends on a deeper comprehension of the molecular processes behind the rapid antidepressant effects of esketamine.

ERK1/2 is a member of the MAPK family, which is involved in cell division and growth, signaling transmission, proliferation and other physiological functions^16^. An earlier study found that ERK1/2 expression is significantly downregulated in the prefrontal cortex of depressed and suicidal individuals^17^. Other investigations into the etiopathogenesis of depression-like behavior have revealed a connection between p-ERK1/2 and depressed symptoms and the ability of antidepressants, such as ketamine, to lessen depressive symptoms by enhancing p-ERK1/2 expression^18^. However, the relationship between esketamine, irisin, and the ERK1/2 signaling pathway in depression remains unclear. In this study, we investigated irisin levels in the hippocampus and peripheral blood of mice with chronic unpredictable mild stress (CUMS) and determined the mechanism of action of esketamine on irisin expression.

## Results

### Esketamine increasing the level of irisin in the hippocampus and peripheral blood of CUMS mice

**Figure 1:**
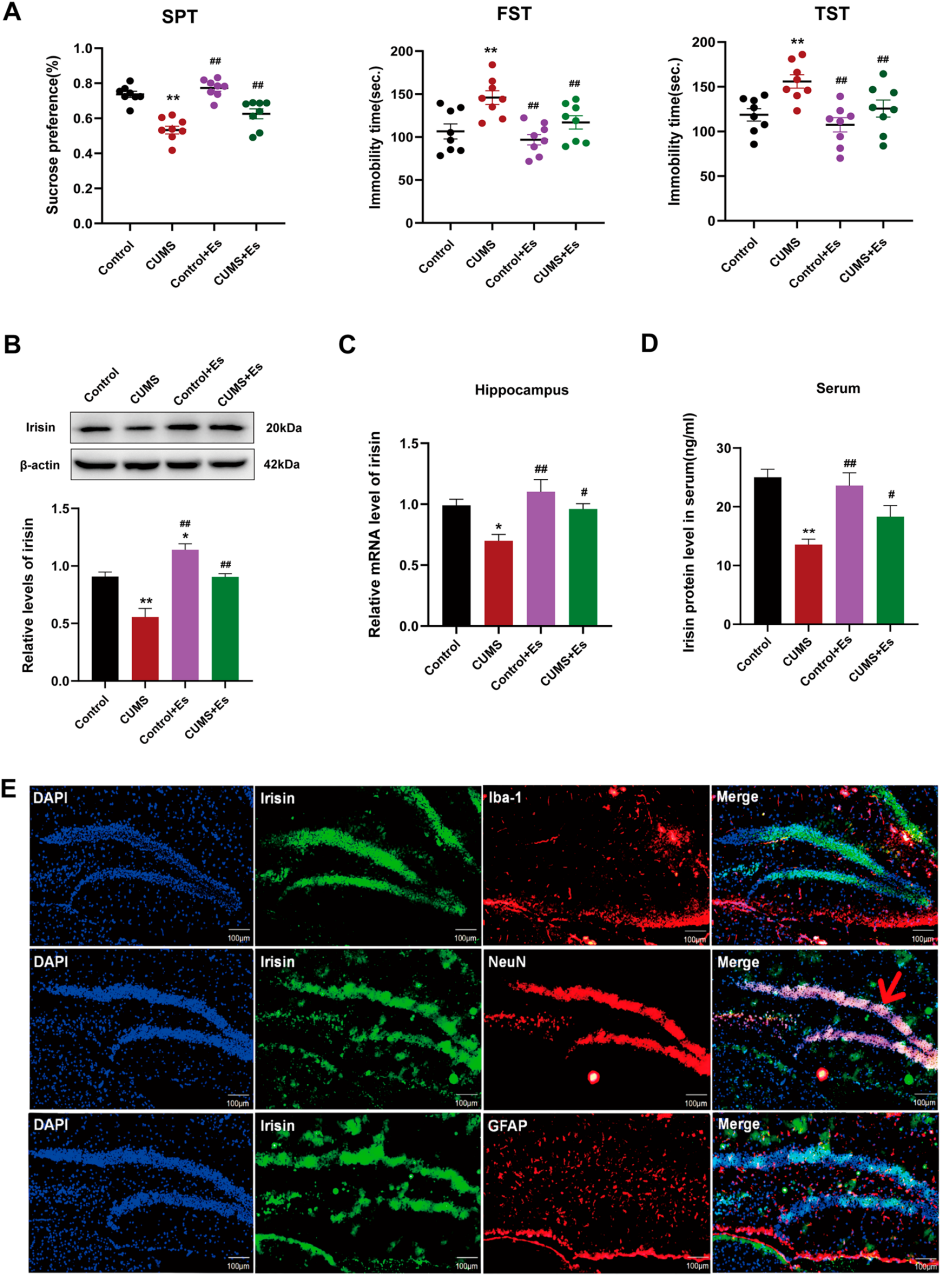
Irisin involved in the antidepressant effect of esketamine. (**A**) SPT (F_[3,28]_ = 30.64, *P* < 0.001), FST (F_[3,28]_ = 11.04, *P* < 0.001) and TST (F_[3,28]_ = 8.98,* P* < 0.001) were performed after a single dose of esketamine (5 mg/kg). N = 8 per group. (**B**) Protein expression level of irisin in the hippocampus (N = 4 per group, F_[3,12]_ = 21.82, *P* < 0.001). (**C**) The irisin mRNA level from the hippocampus (N = 8 per group, F_[3,28]_ = 6.77, *P* = 0.0014). (**D**) Serum irisin protein level by ELISA kit (N = 8 per group, F_[3,28]_ = 12.34,* P* < 0.001). (**E**) Cortical section of the mouse brain was stained to visualize irisin (green) with Iba-1 (red), NeuN (red), or GFAP (red), as indicated to characterize irisin expression in microglia, neurons(arrow symbols), or astrocytes (scale bar = 100 μm). Versus the control group (^*^P < 0.05, ^**^P < 0.01) and the CUMS group (^#^*P* < 0.05, ^##^*P* < 0.01) using one-way ANOVA followed by Bonferroni's post-hoc test.

As shown in Fig. 1, our study found for the first time that esketamine treatment significantly increased irisin levels in the hippocampus (Fig. 1B, *P* < 0.01; Fig. 1C, *P* < 0.05) and peripheral serum of CUMS mice (Fig. 1D, *P* < 0.05). Co-staining of irisin with neuron-specific markers (Neuron), astrocyte markers (GFAP), and microglial markers (Iba-1) showed that irisin was mainly localized to neurons in the hippocampus (Fig. 1E).

### Esketamine regulating irisin expression via PGC-1α

**Figure 2:**
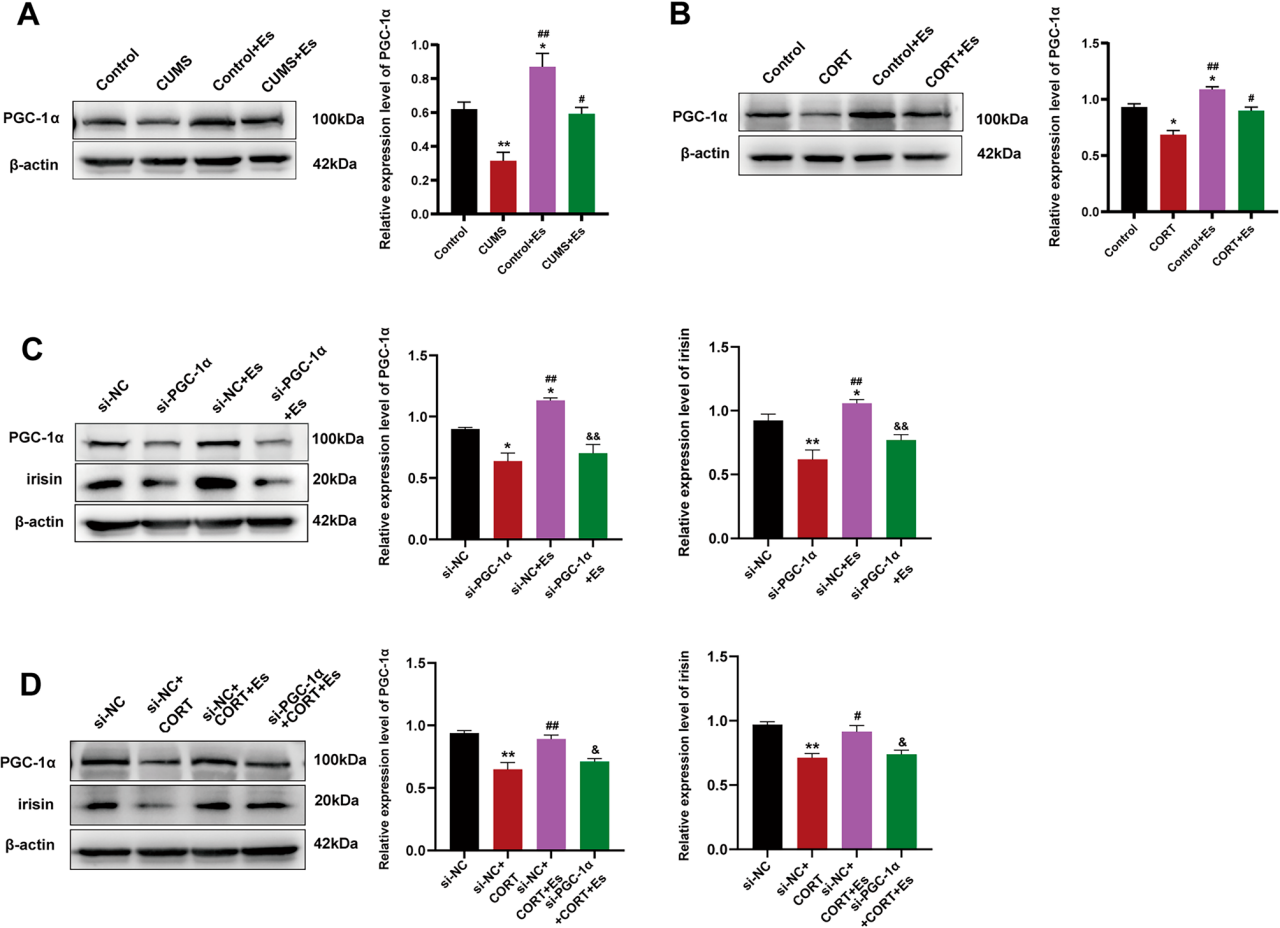
Esketamine regulating the irisin expression via PGC-1α. (**A**–**B**) The expression level of PGC-1α in vivo (F_[3,12]_ = 17.33, *P* < 0.001) or in vitro (F_[3,12]_ = 28.92, *P* < 0.001). N = 4 per group. Versus the control group (^*^P < 0.05, ^**^P < 0.01) and the CUMS or CORT group (^#^*P* < 0.05, ^##^*P* < 0.01) using one-way ANOVA followed by Bonferroni's post-hoc test. (**C**–**D**) HT22 cells were transfected with siRNAs (40 nM) for 24 h, and were subsequently treated with esketamine (25 μM) in the presence or absence of CORT for 24 h. (**C**) Protein levels of PGC-1α (F_[3,8]_ = 19.95, *P* < 0.001) and irisin (F_[3,8]_ = 40.87, *P* < 0.001) were determined by western blot (N = 3 per group). Versus the si-NC group (^*^P < 0.05, ^**^P < 0.01), the si-PGC-1α group (^##^*P* < 0.01), the si-NC + Es group (^&&^*P* < 0.01) using one-way ANOVA followed by Bonferroni's post-hoc test. (**D**) Protein levels of PGC-1α (F_[3,8]_ = 15.75, *P* = 0.001) and irisin (F_[3,8]_ = 13.56, *P* < 0.001) were determined by western blot (N = 3 per group). Versus the si-NC group (^**^P < 0.01), the si-NC + CORT group (^#^*P* < 0.05, ^##^*P* < 0.01), and the si-NC + CORT + Es group (^&^*P* < 0.05) using one-way ANOVA followed by Bonferroni's post-hoc test.

Our data further demonstrated that esketamine treatment enhanced the irisin expression via increasing the expression of PGC-1α (a transcriptional regulator of irisin gene expression in the brain^19^). First, the expression of PGC-1α was reduced in the CUMS and CORT groups (Fig. 2A, *P* < 0.01; Fig. 2B, *P* < 0.05) and increased by esketamine treatment (Fig. 2A–B, *P* < 0.05). Second, the effects of esketamine on increasing the PGC-1α and irisin levels were all diminished by PGC-1α knockdown compared to the control group or CORT group (Fig. 2C, *P* < 0.01; Fig. 2D, *P* < 0.05). The knockdown efficiency of si-PGC-1α was assessed by fluorescence microscopy and western blot (Fig. S1*, P* < 0.01).

### Irisin mediating the antidepressant effect of esketamine via the ERK1/2 signaling pathway

**Figure 3:**
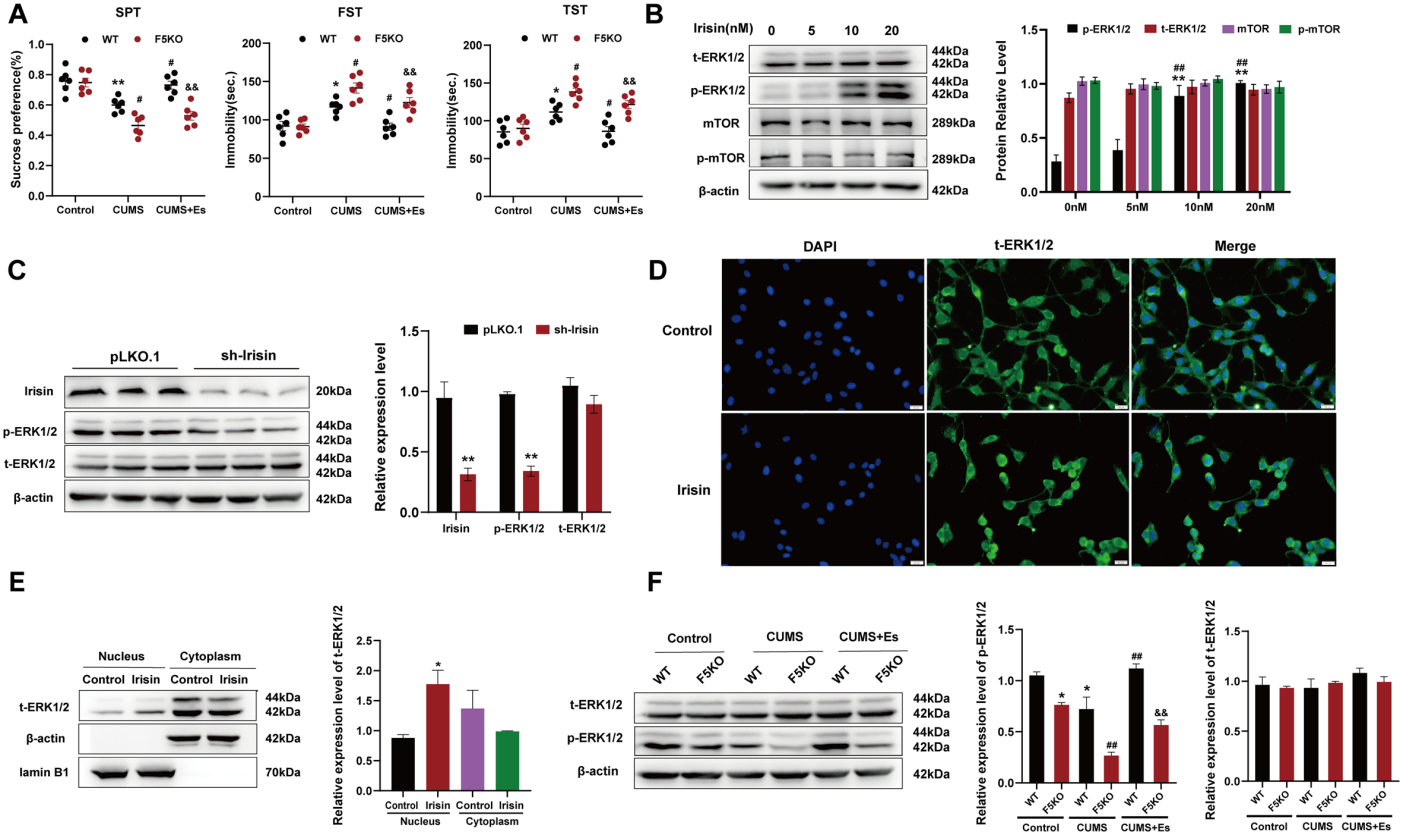
Deletion of FNDC5/irisin attenuating the antidepressant effect of esketamine. (**A**) SPT, FST, and TST were performed after a single dose of esketamine (5 mg/kg). N = 6 per group. SPT—esketamine:F_(2,30)_ = 32.52, *P* < 0.001; F5KO: F_(1,30)_ = 26.71, *P* < 0.001, interaction: F_(2,30)_ = 6.360, *P* = 0.005. FST—esketamine: F_(2,30)_ = 24.37, *P* < 0.001; F5KO: F_(1,30)_ = 17.31, *P* < 0.001; interaction: F_(2,30)_ = 4.820, *P* = 0.0153. TST—esketamine: F_(2,30)_ = 22.11, *P* < 0.001; F5KO: F_(1,30)_ = 23.52, *P* < 0.001, interaction: F_(2,30)_ = 4.078, *P* = 0.0271. Versus the Control-WT group (^*^P < 0.05, ^**^P < 0.01), the CUMS-WT group (^#^*P* < 0.05), and the CUMS + Es-WT group (^&&^*P* < 0.01) using two-way ANOVA followed by Bonferroni's post-hoc test. (**B**) The effect of irisin treatment with different concentrations on the expression of t-ERK1/2 (F_[3,12]_ = 0.7364, *P* = 0.55), p-ERK1/2 (F_[3,12]_ = 22.10, *P* < 0.001), mTOR (F_[3,12]_ = 0.97,* P* = 0.44), and p-mTOR (F_[3,12]_ = 0.65, *P* = 0.61) in HT22 cells treated for 24 h (N = 4 per group). Versus the control group (^**^P < 0.01) and HT22 cells with 5 nM irisin treatment (^##^*P* < 0.01) using one-way ANOVA followed by Bonferroni's post-hoc test. (**C**) HT22 stable low expression cell line was established by lentivirus infection, and pLKO.1 stable expression cell line was used as a control. Relative expression of irisin (t = 5.120, *P* < 0.01), t-ERK1/2 (t = 1.555, *P* = 0.1950), and p-ERK1/2 (t = 5.566, *P* < 0.01) were detected by western blot (N = 3 per group). Versus the pLKO.1 group (^**^P < 0.01) using Student’s t-test. (**D**) Immunofluorescent staining for the subcellular location of t-ERK1/2 induced by 10 nM irisin treatment for 24 h (N = 3 per group). (**E**) Nucleocytoplasmic separation experiments for demonstrating the localization of t-ERK1/2 (N = 3 per group, F_[3,8]_ = 4.44, *P* = 0.04). Versus the control group of the nucleus (^*^P < 0.05) using one-way ANOVA followed by Bonferroni's post-hoc test. **F**: The expression of t-ERK1/2 and p-ERK1/2 protein in the hippocampus of WT and F5KO mice (N = 4 per group, esketamine: F_[2,18]_ = 41.69, *P* < 0.001; F5KO: F_[1,18]_ = 53.67, *P* < 0.001; interaction: F_[2,18]_ = 10.13, *P* = 0.0011). Versus the Control-WT group (^*^P < 0.05), the CUMS-WT group (^##^*P* < 0.01), and the CUMS + Es-WT group (^&&^*P* < 0.01) using two-way ANOVA followed by Bonferroni's post-hoc test.

As shown in Fig. 3, we confirmed for the first time that irisin mediated the antidepressant effect of esketamine via the ERK1/2 signaling pathway. Firstly, the antidepressant effects of esketamine were attenuated in F5KO mice (Fig. 3A, *P* < 0.01). Secondly, our data revealed that 10 or 20 nM irisin treatment increased the p-ERK1/2 expression levels in HT22 cells (Fig. 3B, *P* < 0.01). When irisin gene expression was reduced in HT22 cells using lentiviral-mediated shRNA knockdown, p-ERK1/2 protein expression was significantly lower (Fig. 3C, *P* < 0.01). Thirdly, IF staining and immunoblotting showed that irisin treatment resulted in the translocation of t-ERK1/2 from the cytoplasm to the nucleus (Fig. 3D; Fig. 3E, *P* < 0.05). Finally, the immunoblotting results showed that the CUMS-induced decrease in p-ERK1/2 expression in the hippocampus was further reduced in F5KO mice (Fig. 3F, *P* < 0.01), and the esketamine-induced increase in p-ERK1/2 expression was significantly attenuated in F5KO mice (Fig. 3F, *P* < 0.01).

## Discussion

Our study demonstrated that FNDC5/irisin has an important role in the rapid antidepressant effects of esketamine. Upregulation of FNDC5/irisin may thus represent a potential therapeutic target for depression. The functional relationship between esketamine and FNDC5/irisin is clarified by this study, which also offers a fresh perspective on how to design a proactive plan and efficient MDD therapy.

Although multiple mechanisms may exist, the mechanism underlying the antidepressant action of esketamine has not been fully elucidated. Our results showed that esketamine may exert the antidepressant effects by regulating the irisin level. Esketamine increased irisin mRNA and protein expression levels in the hippocampus, and further research found that irisin was mainly localized in hippocampal neurons, suggesting that esketamine may exert the antidepressant effect through directly regulating the irisin biogenesis in hippocampal neurons and indirectly affecting the circulatory irisin level in peripheral blood. Studies have actually demonstrated that peripheral irisin may pass the blood–brain barrier (BBB) and penetrate the central nervous system where it might operate as a protective agent^20^. Notably, a recent study showed that FNDC5 mRNA levels in the hippocampus of three models (lipopolysaccharide-, chronic CORT-, and social isolation-induced models) were not significantly changed compared to controls, and ketamine did not significantly affect FNDC5 mRNA^21^. This discrepancy may be explained by several factors, including different animal models of depression, different concentrations and durations of drug action, and drug model dependence. To further verify the regulatory relationship between esketamine and irisin, we used global F5KO mice. Our data showed that F5KO mice developed more severe depression-like behaviors than WT mice and attenuated the antidepressant effects of esketamine. Clinical studies have reported a significant negative correlation between irisin levels and depression scale scores in patients^22,23^. Correspondingly, preclinical studies have found that irisin levels in the rat prefrontal cortex gradually decrease with increasing duration of CUMS stimulation. Furthermore, irisin levels in the FST showed a negative link with immobility time, but a clear positive correlation with sucrose preference^10^. These results suggest that a decrease in irisin exacerbates depression in rats and patients, which is consistent with the results of our study. This phenomenon may be explained by the fact that a reduction in irisin makes subjects more sensitive to external stimuli. However, our study only showed that irisin was correlated with more severe depression-like phenotypes and the antidepressant effects of esketamine. Thus, further studies, such as irisin rescue experiments, are warranted to confirm the cause-effect relationship between irisin and disease phenotypes.

PGC-1α was first identified as a transcriptional coactivator of oxidative metabolism and mitochondrial biogenesis in brown fat^24^. Subsequent studies demonstrated that PGC-1α was a transcriptional regulator of irisin gene expression in the brain and played an important role in depression^19,25^. In this study, we found that the expression of PGC-1α was reduced in the CUMS and CORT groups and increased following esketamine treatment, which was consistent with the irisin expression. PGC-1α knockdown can partially prevent esketamine-induced increases in irisin expression. Similar results were observed in a neuronal CORT model. These data suggested that esketamine may regulate the expression level of irisin through PGC-1α. Given that PGC-1α may be involved in the antidepressant effects of esketamine, it is necessary to further verify the changes in mouse behavior after regulating PGC-1α expression at the animal level in the future. Sirtuin-1 (sirt1) was shown to have the effect of deacetylation of downstream PGC-1α and modulated its activities^26^. Preclinical investigations have indicated the potential role of sirt1 in anxiety-like and depressive-like behavior^27^. Additionally, clinical studies have observed a positive association between sirt1 downregulation and depression in patients^28^. Consequently, whether the involvement of sirt1/ PGC-1α in the antidepressant effect of esketamine remains uncertain and needs further investigation in subsequent research.

It has been demonstrated that irisin was closely related to the ERK1/2 pathway^29–31^. Our data showed that irisin (10 or 20 nM) treatment activated the ERK1/2 pathway. Studies have reported that the ERK1/2 pathway is activated and ERK1/2 is translocated to the nucleus, where ERK1/2 activated specific transcription factors to regulate gene transcription^32^. Our IF and immunoblotting results also confirmed the translocation to the nucleus. It is currently unclear how irisin activates ERK/1/2 pathway, irisin has only been reported to bind to integrin αV receptors and activate downstream pathways in bone, fat and human osteoblasts^33,34^. The specific cellular receptor of irisin in the brain has not yet been identified, thus this aspect limits the study of downstream signaling mechanisms and further research is needed.

However, it is unclear how the ERK1/2 signaling pathway is involved in antidepressant effects. The ERK1/2 pathway in the brain areas associated with severe depression is sensitive to chronic stress, and ERK1/2 activity is decreased in the prefrontal cortex and hippocampus of suicide participants, according to mounting data^35^. Previous research found that the transcription factors EIK-1, CREB, c-Jun, and Fos are downstream targets of ERK1/2, which affect neuronal excitation by regulating long-term potentiation, axon growth, and the release of neurotransmitters from the presynaptic membrane, and finally exert antidepressant effects by participating in synaptic protein synthesis and regulating synaptic structural plasticity^36^. Moreover, ketamine increases the expression of BDNF, whose transcription is dependent on CREB, suggesting that the BDNF-ERK pathway may be involved in synaptic plasticity, regulating neuronal survival, and axonal growth to exert antidepressant effects^32^. Hence, the ERK1/2 signaling pathway may be involved in improving synaptic plasticity, exerting antidepressant effects.

**Figure 4:**
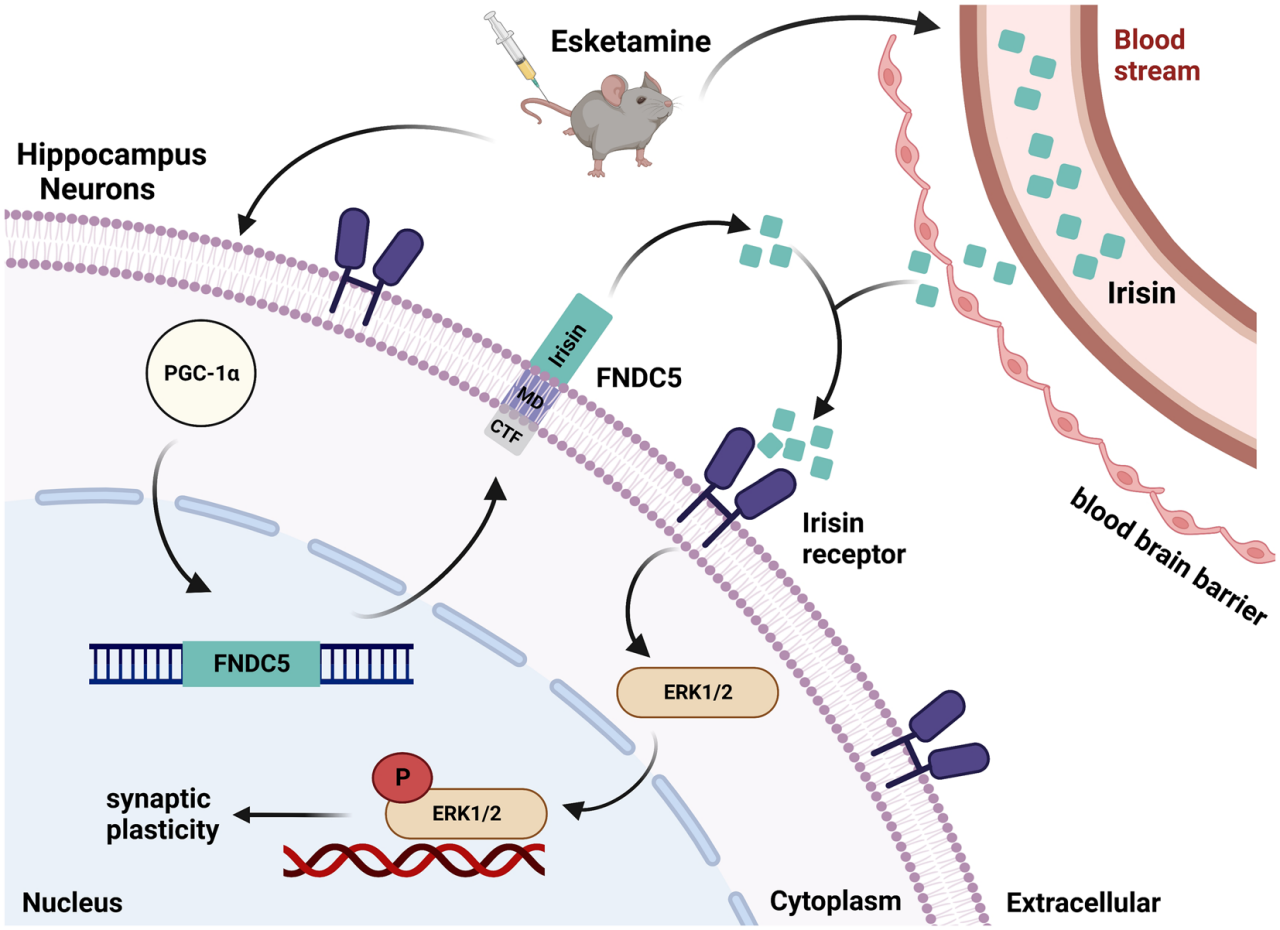
Mechanism diagram. A schematic diagram depicting irisin mediated the antidepressant effect of esketamine via ERK1/2 signaling pathway.

In conclusion, this is the first study to demonstrate that esketamine upregulated the expression of PGC-1α, which was accompanied by the increase of irisin gene expression, resulting in t-ERK1/2 translocation into the nucleus and producing rapid and long-lasting antidepressant-like actions (Fig. 4).

## Methods

### Ethics statement

The animal experiment was carried out in strict accordance with the Guidelines for the China Laboratory Animal Science Association and the regulations of the Animal Ethics Committee of the Affiliated Hospital of Southwest Medical University (Approval number: SWMU20220087). All methods were carried out in accordance with relevant guidelines and regulations. This study was also carried out in compliance with the ARRIVE guidelines.

### Animals and treatments

Male C57BL/6 J mice (20.0–26.0 g, 6–8 weeks old) were acquired from the Chinese company Chongqing Tengxin and randomly allocated to experimental groups. Global FNDC5/irisin KO(F5KO) mice were purchased from Cyagen Biosciences Inc. (China). The mice were born at an approximate Mendelian ratio with no gross abnormalities. All of the animals were kept in enclosures with constant humidity, light/dark cycle, and temperature.

**Figure 5:**
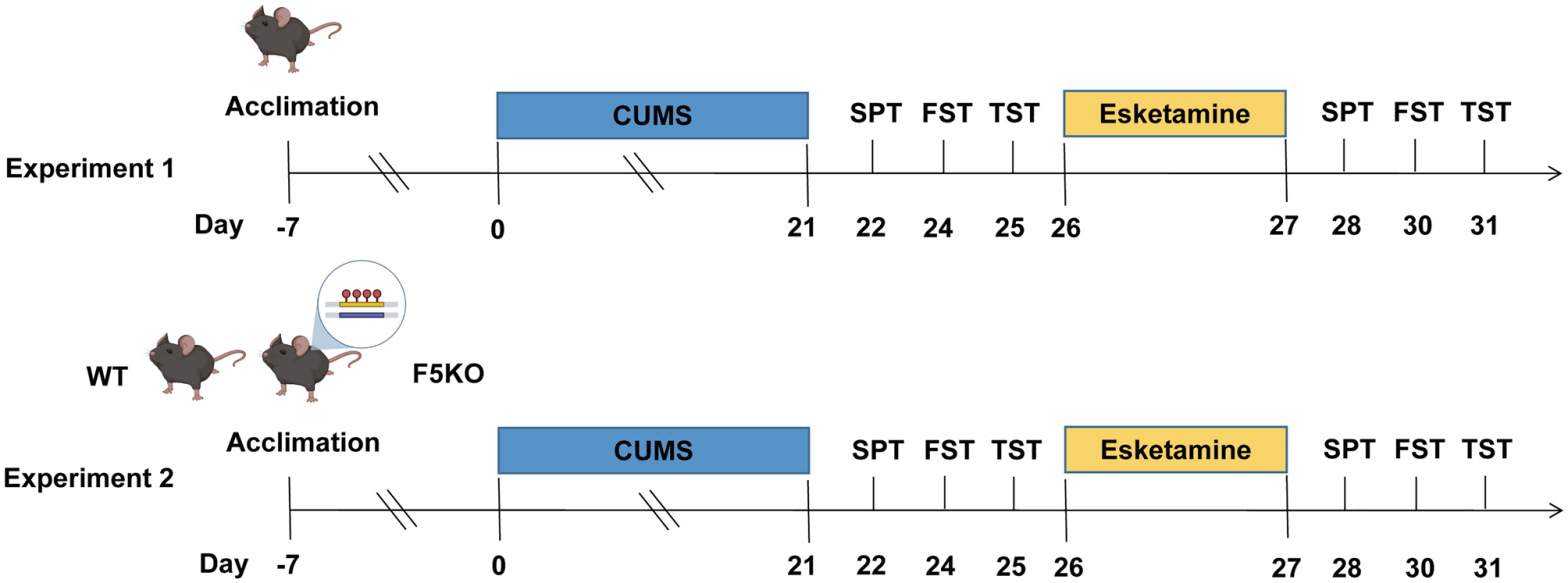
Experimental design. CUMS: chronic unpredictable mild stress; SPT: sucrose preference test; FST: forced swim test; TST: tail suspension test; WT: wild type; F5KO: FNDC5/irisin-knock out.

Mice were randomly assigned to the normal control group without any treatment and the test group with CUMS. We used a previously validated protocol for inducing CUMS in the mice^37^.The experimental stressors were delivered to the mice in a random order over the course of 21 days, preventing the administration of any one stressor on two consecutive days and no more than twice a week. The experimental flow diagram is exhibited in Fig. 5. Significant variations in depression-like behaviors obtained on experimental days 21–25 compared to those of the control group were used to judge whether the CUMS model had been successfully established. Thereafter, the mice were grouped and each group was injected with 100 μL corresponding drugs via the tail vein. Esketamine (H20193336, Minsheng Pharma Holdings, China) was dissolved in 0.9% saline. Experiment 1: Control (received 0.9% saline), CUMS (received 0.9% saline), Control + Es (received esketamine, 5 mg/kg), CUMS + Es (received esketamine, 5 mg/kg), n = 8/group. Experiment 2: Control (wild-type [WT] or F5KO mice received 0.9% saline), CUMS (WT or F5KO mice received 0.9% saline), and CUMS + Es (WT or F5KO mice received esketamine, 5 mg/kg), n = 6/group.

### Behavioral tests

Behavioral tests, including the sucrose preference test (SPT), tail suspension test (TST), and forced swim test (FST), were conducted after CUMS treatment. The same rater completed each test between 9:00 and 17:00 in a sound-attenuated space with low-intensity lighting, and each test was graded. Mice were habituated to the room for at least 3 h before testing.

#### SPT

Mice were continuously exposed to 2 bottles for 24 h, one containing 100 mL of normal water and the other 100 mL of 2% sucrose solution, and their locations were exchanged after 12 h. Before the final test, mice were deprived of water and chow for 12 h and put in separate cages. They had free access to the 2 bottles for 24 h, with the locations swapped after 12 h. The sucrose preference (%) was calculated as the weight of sucrose intake over the total weight of fluid intake.

#### TST

Mice were individually suspended by the tail to a vertical bar on the top of a box (15 × 15 × 30 cm), fixed approximately 1 cm from the tail tip, and kept 15 cm above the bottom. Each test session lasted for 6 min and was recorded. The first 2 min were used as the habituation phase, while the following 4 min were used to record the amount of time spent hanging passively with no movement.

#### FST

Individual mice were dropped into a cylinder with 20 cm in diameter and 25 cm in height, containing 15 cm of water at 23–25 °C. The water was changed after each trial. The mice developed an immobile posture, which was characterized by motionless floating in the water with just the required movements to maintain their heads above the water, after strong activity during the first 2 min. The entire procedure lasted for 6 min, and the final 4 min of immobility were recorded.

### Cell culture and treatments

Mouse hippocampal neuronal cells, HT22, were obtained from the Stem Cell Bank, Chinese Academy of Sciences. The cells were cultured in DMEM (GBICO, USA) supplemented with 10% fetal bovine serum (PEAK SERUM, US), penicillin (100 U/mL), and streptomycin (100 µg/mL) in a 5% CO2 atmosphere at 37 °C. Corticosterone (CORT) (BD122290-250 mg, Bidepharm, China) was dissolved in absolute ethanol. Cultured HT22 cells were incubated with 125 μM CORT for 24 h, which mimicked depression due to a continuous increase in glucocorticoids, leading to an imbalance in the regulation of hippocampal neuronal plasticity^38^.To detect the effect of esketamine and irisin in vitro, the HT22 cells were treated with 25μMesketamine or different concentrations of irisin in the presence or absence of CORT for 24 h.

### Cell transfection

Predesigned and validated small interfering RNAs (siRNAs) specific for PGC-1α (sense: 5′-CCAAGACUCUAGACAACUATT-3′; antisense: 5′-UAGUUGUCUAGAGUCUUGGTT-3′), together with control siRNA (FITC Conjugate)-A (si-NC), were obtained from Santa Cruz Biotechnology. In accordance with the manufacturer's instructions, transfection was carried out using the jetPRIME transfection reagent (Polyplus, France).The cells were examined 24 h after transfection.

The gene sequences of irisin were obtained from NCBI and a pair of short hairpin (sh-RNA) primers were designed according to the gene sequences of irisin: forward 5′-CCGGGGATGTCCTGGAGGATGAAGTCTCGAGACTTCATCCTCCAGGACATCCTTTTTG-3′ and reverse 5′-AATTCAAAAAGGATGTCCTGGAGGATGAAGTCTCGAGACTTCATCCTCCAGGACATCC-3′. The interference segment was ligated into the pLKO.1-TRC cloning vector (pLKO.1) to construct an irisin knockdown vector (pLKO.1-sh-irisin). To generate the recombinant lentivirus LV-sh-irisin, 293 T cells were co-transfected with the pLKO.1-sh-irisin plasmid (2 μg), VSVG plasmid (0.5 μg), and DVPR plasmid (1.5 μg) using the jetPRIME transfection reagent (8 μL). Lentivirus supernatants were collected 48 and 72 h after transfection. Moreover, stably transfected cells were selected using 5 μg/mL puromycin 72 h after infection with the lentiviral supernatant, and HT22 cells with stable low irisin expression were validated by immunoblotting.

### Western blot and enzyme-linked immunosorbent assay (ELISA) analysis

The radio-immunoprecipitation assay buffer (Beyotime, Shanghai, China) was used to separate the proteins employed in the western blot analysis, and the Enhanced BCA Protein Assay Kit (Beyotime, Shanghai, China) was used to measure their concentration. Equal amounts of lysate samples (20 µg/lane) were divided using 12% SDS-PAGE and then transferred to nitrocellulose-flter (NC) membranes with a thickness of 0.22 μm. The membranes were blocked for 1 h at room temperature with 5% skimmed milk and then incubated with primary antibodies against FNDC5 (Cell Signaling Technology, 1:1000), ERK1/2 (Beyotime, 1:1000), phospho-ERK1/2 (Beyotime, 1:1000), mTOR (Beyotime, 1:1000), and phospho-mTOR (Ser2448) (Cell Signaling Technology, 1:1000) at 4 °C overnight. Afterward, the membranes were washed three times with 1 × TBST for 10 min and incubated with horseradish peroxidase-conjugated AffiniPure goat anti-mouse IgG (H + L) (Proteintech, 1:5000) or HRP-conjugated AffiniPure goat anti-rabbit IgG (H + L) (Proteintech, 1:5000) for 1 h at room temperature. After washing with 1 × TBST three times for 10 min each, the bands were visualized using the ECL Western Blotting Substrate (Solarbio, Beijing, China). The gray values were quantified using the ImageJ program using β-actin as an internal reference. Serum irisin levels were detected in mouse samples via an immunoassay kit (Mouse irisin ELISA kit, ZCIBIO, China), and the protocol was adopted according to the manufacturer’s instructions.

### RNA extraction and quantitative RT-PCR

Following the manufacturer’s instructions, total RNA was extracted from cultures or animal tissues using TRIzol reagent (Ambion, USA). 1 µg of total RNA was used for cDNA synthesis using the HiScript III 1st Strand cDNA Synthesis Kit (+ gDNA wiper) (Vazyme, China). Quantitative expression analysis of the target genes was performed using a QuantStudio 5 Applied Biosystems (Thermo Fisher Scientific, USA) real-time PCR system with PowerUp SYBR Green Master Mix (Thermo Fisher Scientific, USA). The fold change in the expression of the target gene was determined using the comparative Ct approach (2-ΔΔCt method) with β-actin as the housekeeping gene. The primer sequences utilized for qRT-PCR are listed in Table 1.

**Table 1:** qRT-PCR primers.

Species	Genes	Forward primer	Reverse primer
Mus musculus	FNDC5/irisin	TCCTGGAGGATGAAGTGGTC	TGAAGAGCACAGGCTCACTG
	β-actin	CCTAAGGCCAACCGTGAAAA	GAGGCATACAGGGACAGCACA

### Immunofluorescence (IF) staining

Mouse brain frozen sections (10 μm) and cells were fixed in 4% PFA for 30 min, then followed by three washes in phosphate-buffered saline (PBS), permeabilized with 0.5% Triton X-100 (Beyotime, Shanghai, China) in PBS for 20 min, blocked in 3% BSA (Solarbio, Beijing, China) for 1 h, and incubated with primary antibodies FNDC5 (Affinity, 1:200), GFAP (Beyotime, 1:100), NeuN (CST, 1:500), Iba-1 (abcam, 1:100) or ERK1/2 (Beyotime, 1:100) overnight at 4 °C. The binding antibodies were identified using a 1:500 dilution of AlexaFluor488 or AlexaFluor568 antibody for 1 h at room temperature, followed by 5 min of staining with DAPI (Solarbio, Beijing, China). A fluorescent microscope (OLYMPUS ix83, Japan) was used to acquire the images.

### Statistical analysis

Statistical analyses were performed using the Graph Pad Prism 7 software and significance was determined using Student’s t-test for comparisons between two groups. Furthermore, one-way analysis of variance (ANOVA) and two-way ANOVA followed by Bonferroni's post-hoc test were utilized for multi group comparisons. All data are presented as the mean ± SEM. Statistical analyses performed for different experiments are described in the respective figure legends, and the results were considered significant at* P* < 0.05.

### Supplementary Information

Supplementary Information 1.

Supplementary Figure S1.

## Data Availability

The datasets generated during and/or analyzed during the current study are available from the corresponding author upon reasonable request.
