# Supplementary figures and images for: Exploring the role of esketamine in alleviating depressive symptoms in mice via the PGC-1α/irisin/ERK1/2 signaling pathway
Source: Sci Rep. 2023 Oct 3;13:16611. doi: 10.1038/s41598-023-43684-9 (PMC10547795; doi:10.1038/s41598-023-43684-9)

**Figure1-B**

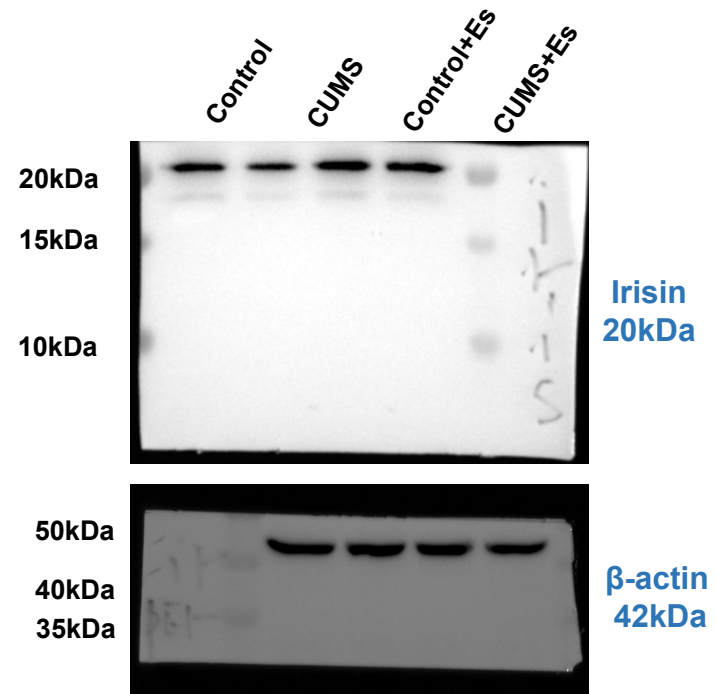

Figure2-A

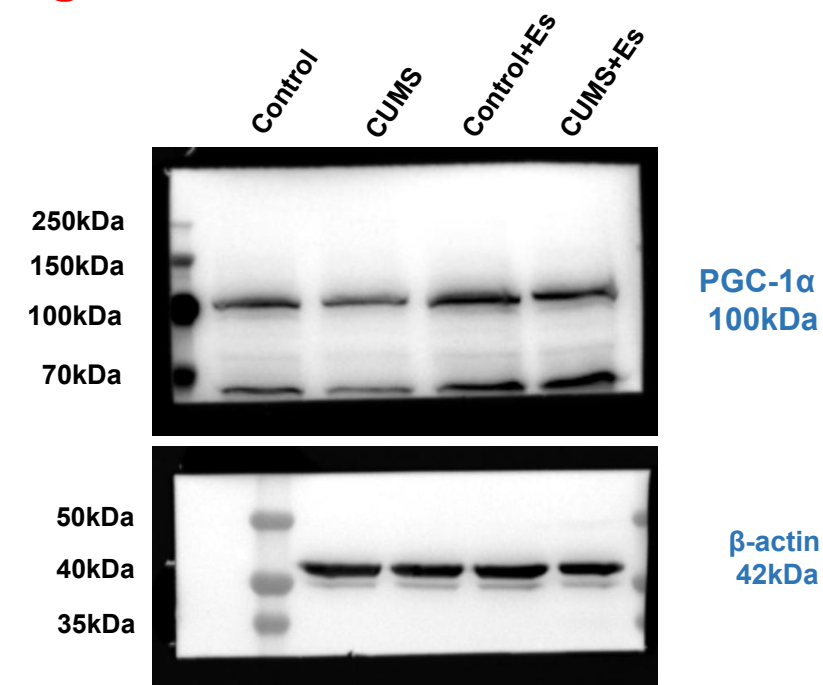

Figure2-B

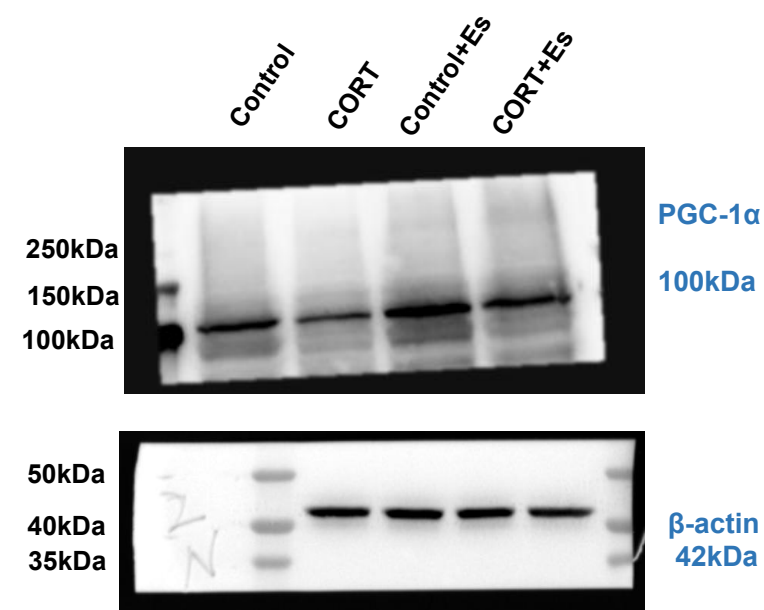

Figure2-C

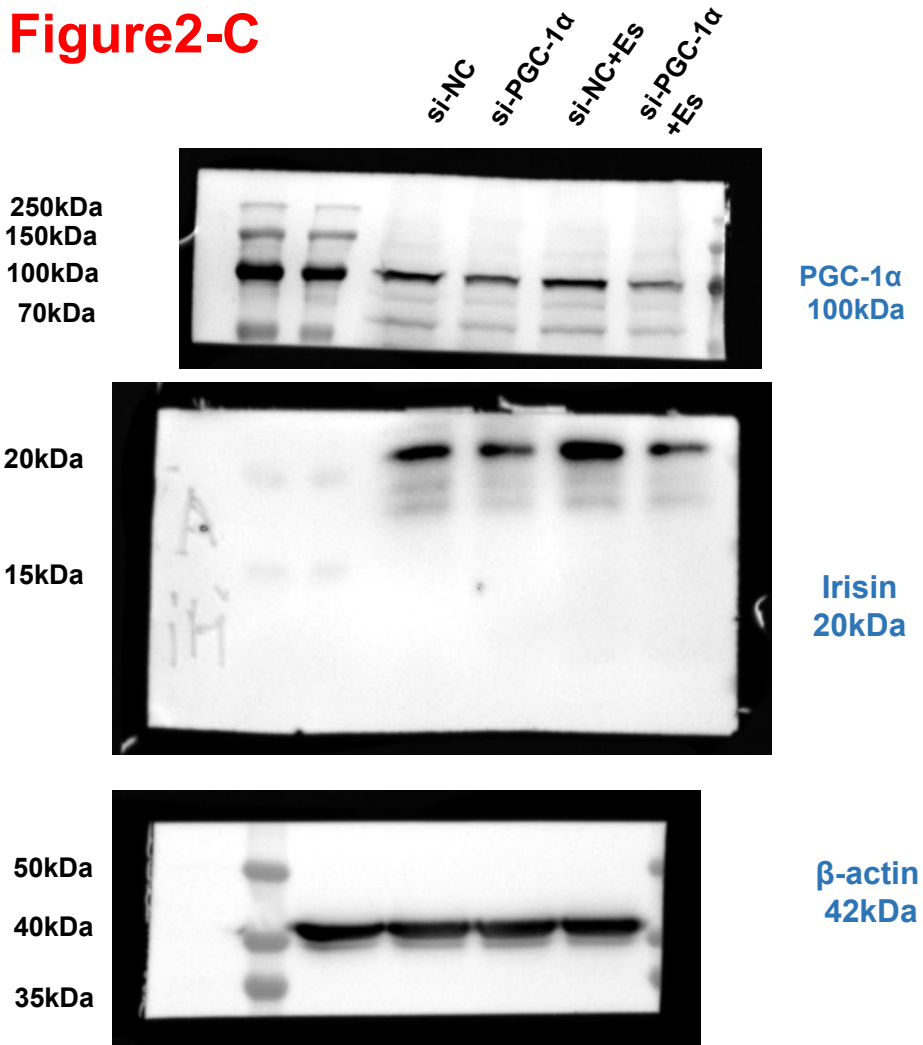

Figure2-D

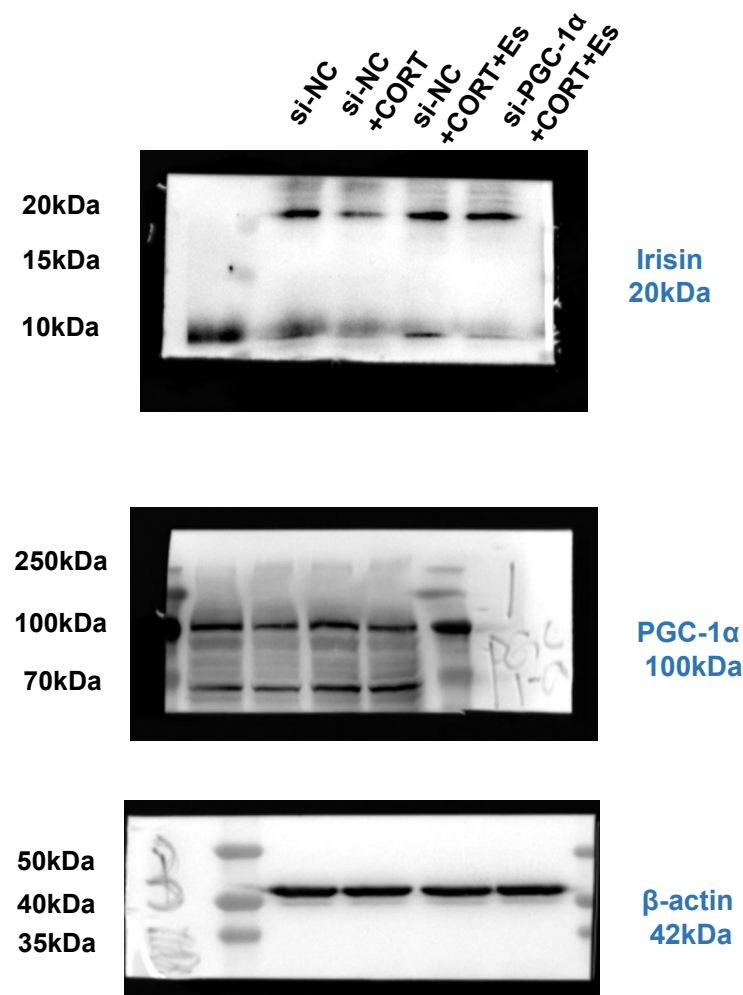

Figure3-B

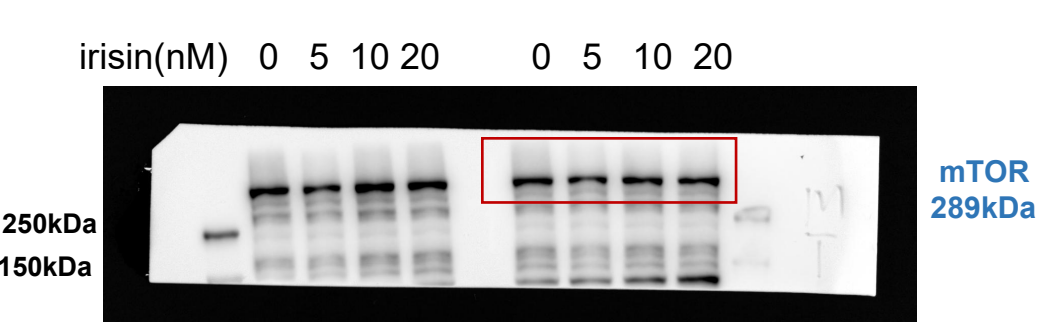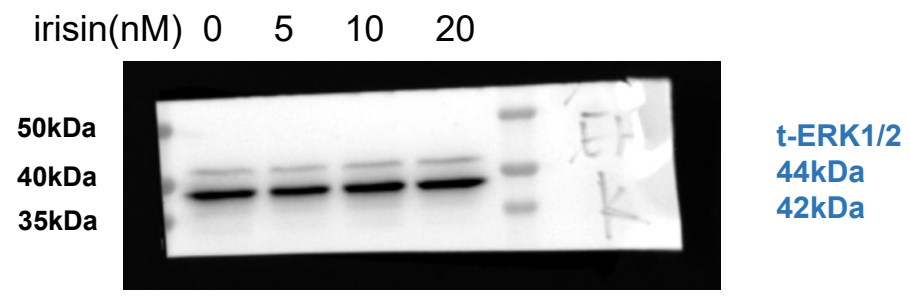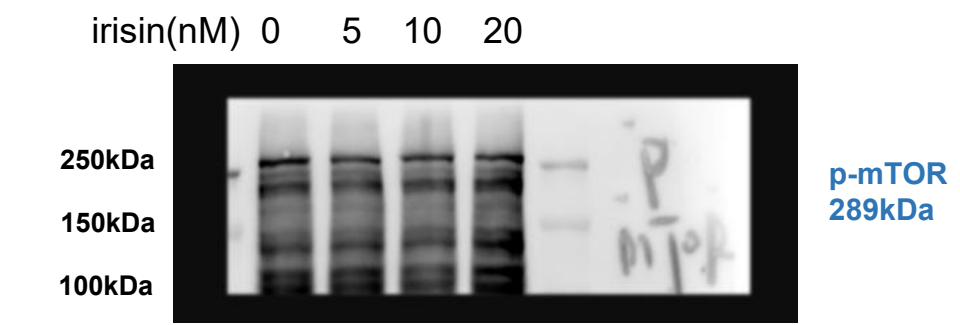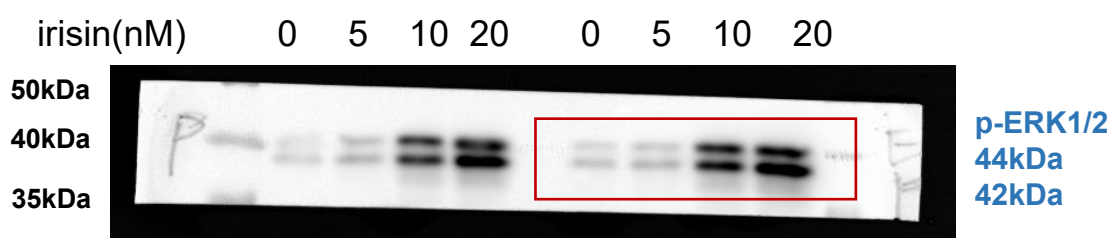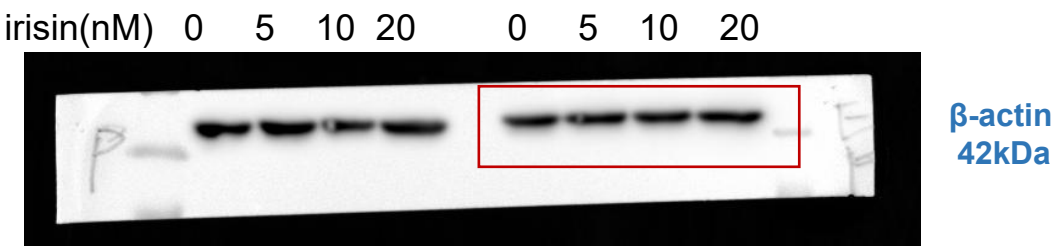

Figure3-C

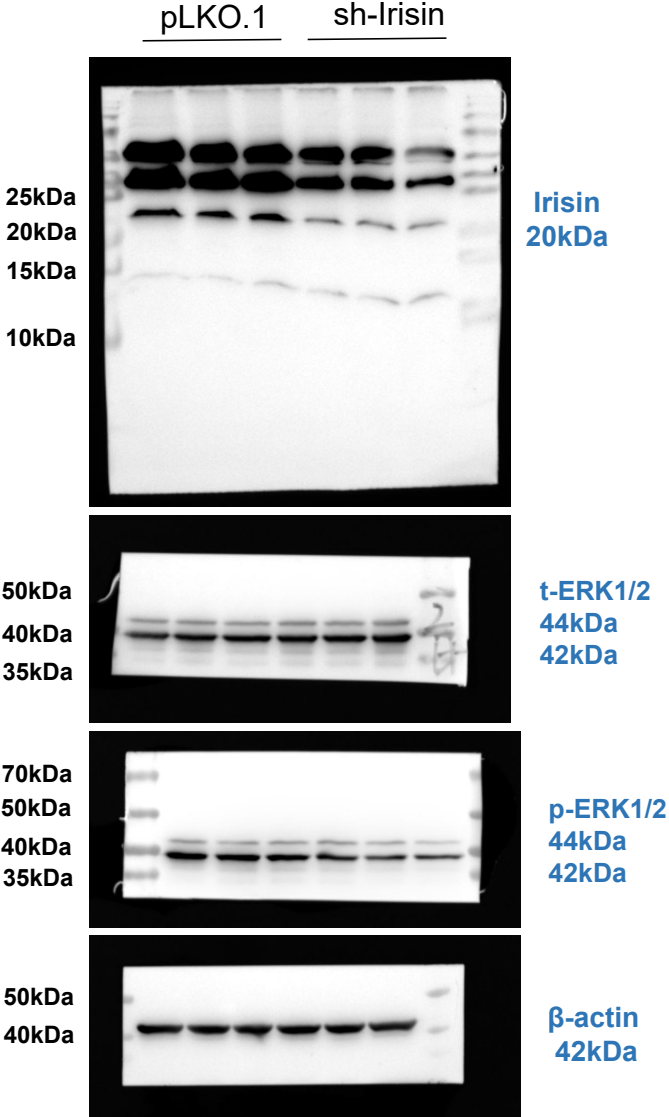

Figure3-E

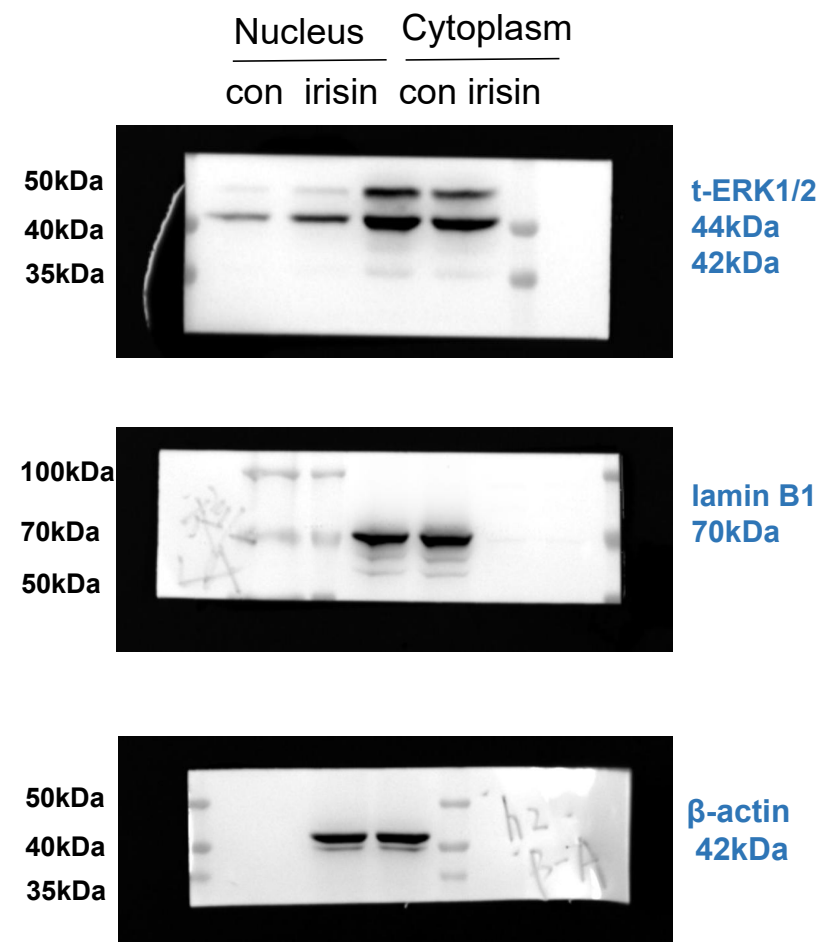

Figure3-F

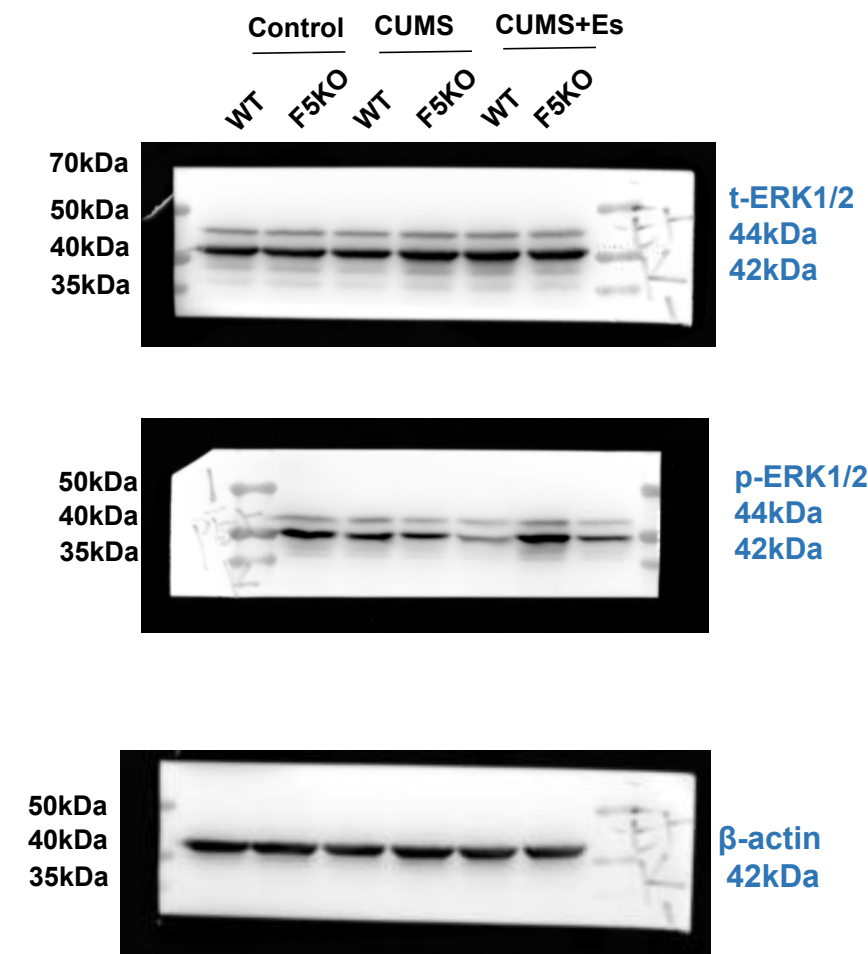

Figure S1

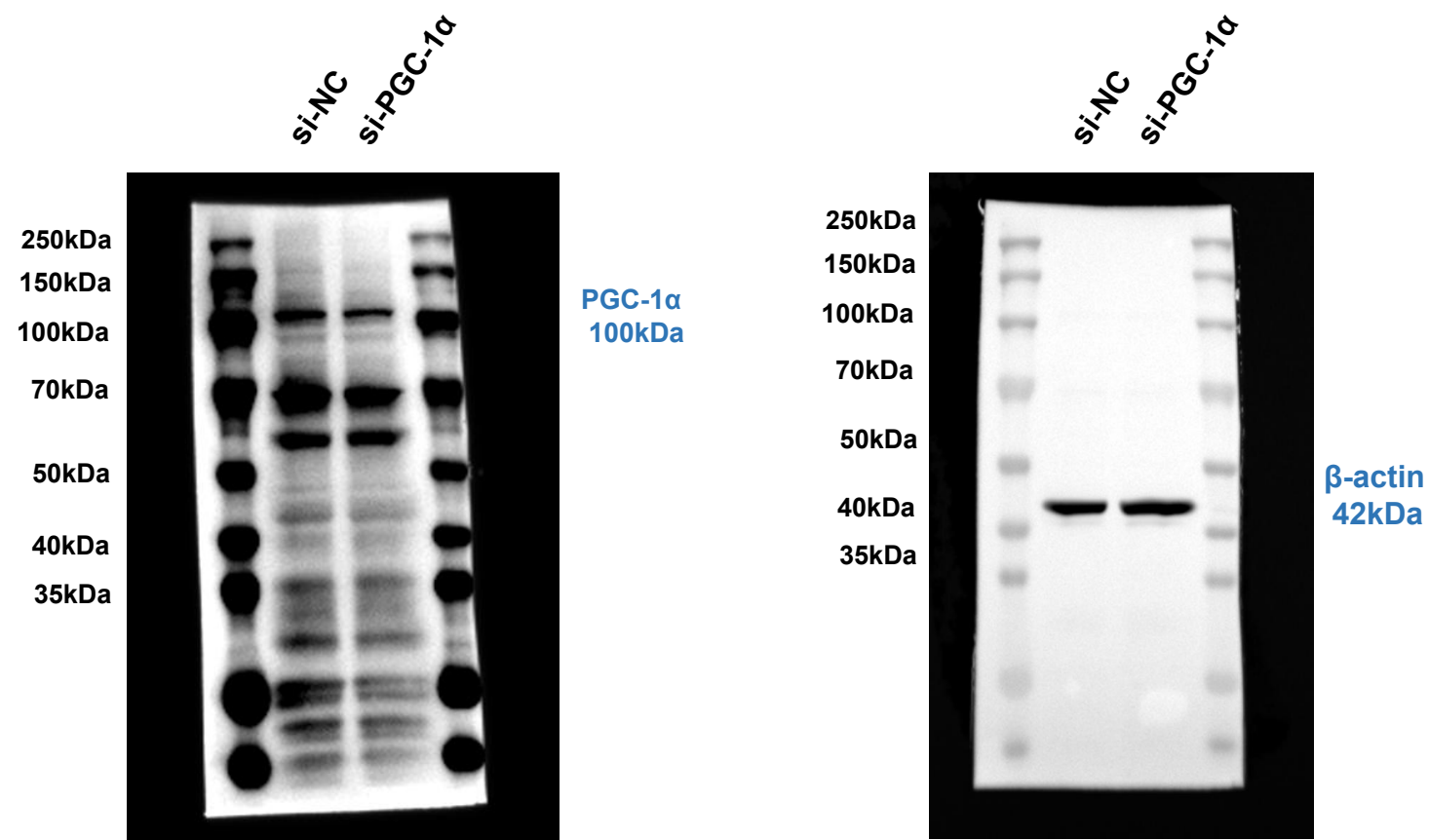

Supplement: Supplementary file 1 — Supplementary Information 1. [file 41598_2023_43684_MOESM1_ESM.pdf]
