# Supplementary material for: Exploring the role of esketamine in alleviating depressive symptoms in mice via the PGC-1α/irisin/ERK1/2 signaling pathway
Source: Sci Rep. 2023 Oct 3;13:16611. doi: 10.1038/s41598-023-43684-9 (PMC10547795; doi:10.1038/s41598-023-43684-9)
Supplement: Supplementary file 2 — Supplementary Figure S1. [file 41598_2023_43684_MOESM2_ESM.docx]

***Supplementary Figures***

**Figure S1**

**
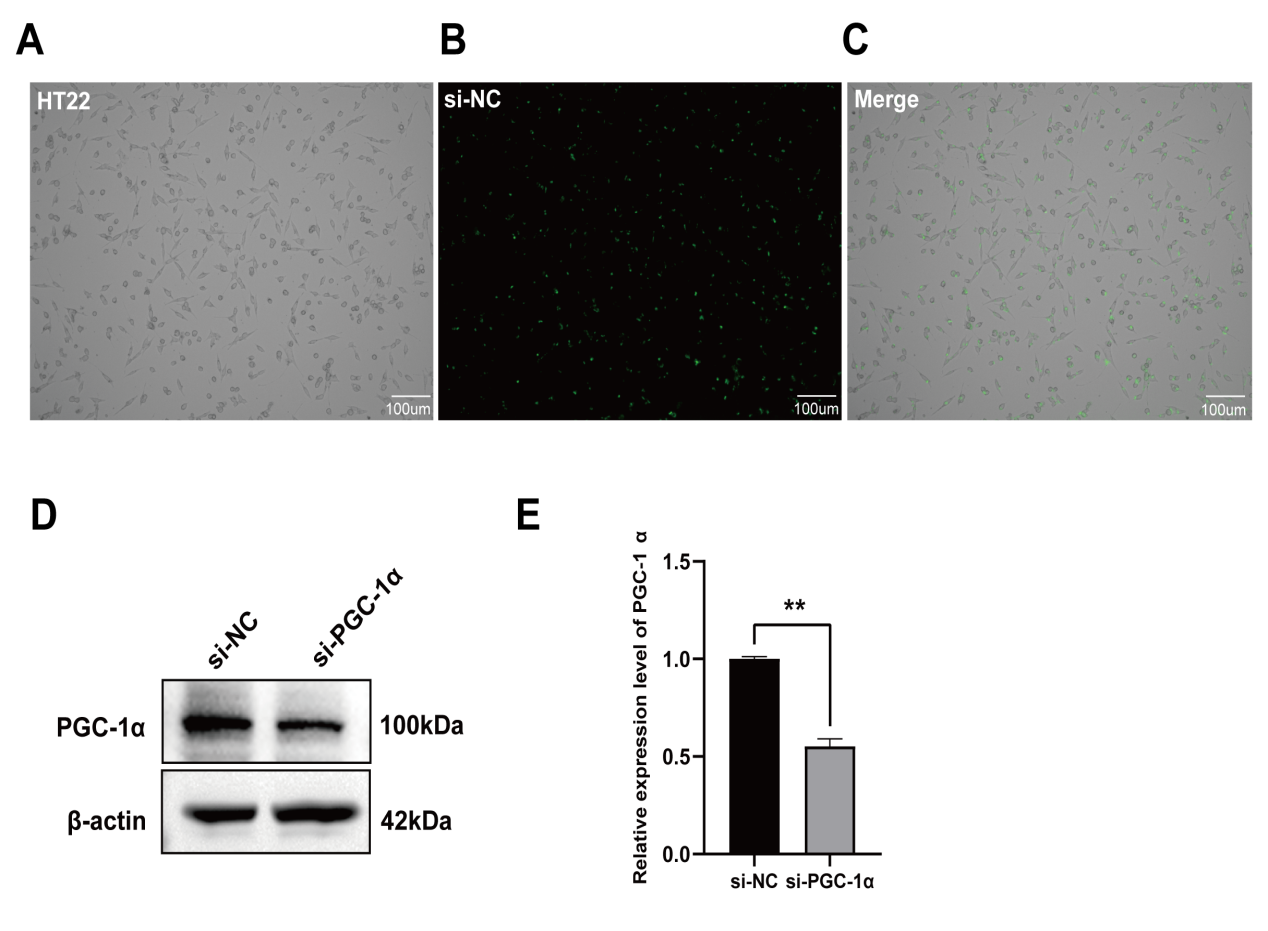
**

**Fig. S1 Validation of knockdown efficiency of PGC-1α in HT22 cells.** **A**–**C:** Control siRNA (FITC Conjugate-A) (si-NC) transfection efficiency was confirmed by fluorescent microscopic observations. Scale bar=100μm. **D**–**E:** Relative expression of PGC-1α in HT22 cells after si-PGC-1α transduction for 24 h as determined by western blot (N=3 per group). Versus the si-NC group (t=11.00, ^**^*P* < 0.01) using Student’s t-test.
